# The effect of race/ethnicity on cancer-specific mortality after salvage radical prostatectomy

**DOI:** 10.3389/fonc.2022.874945

**Published:** 2022-08-19

**Authors:** Mike Wenzel, Christoph Würnschimmel, Luigi Nocera, Claudia Colla Ruvolo, Benedikt Hoeh, Zhe Tian, Shahrokh F. Shariat, Fred Saad, Alberto Briganti, Markus Graefen, Felix Preisser, Andreas Becker, Philipp Mandel, Felix K. H. Chun, Pierre I. Karakiewicz

**Affiliations:** ^1^ Department of Urology, University Hospital Frankfurt, Goethe University Frankfurt, Frankfurt, Germany; ^2^ Cancer Prognostics and Health Outcomes Unit, Division of Urology, University of Montréal Health Center, Montréal, QC, Canada; ^3^ Martini-Klinik Prostate Cancer Center, University Hospital Hamburg-Eppendorf, Hamburg, Germany; ^4^ Department of Urology and Division of Experimental Oncology, URI, Urological Research Institute, IRCCS San Raffaele Scientific Institute, Milan, Italy; ^5^ Department of Neurosciences, Reproductive Sciences and Odontostomatology, University of Naples Federico II, Naples, Italy; ^6^ Department of Urology, Comprehensive Cancer Center, Medical University of Vienna, Vienna, Austria; ^7^ Departments of Urology, Weill Cornell Medical College, New York, NY, United States; ^8^ Department of Urology, University of Texas Southwestern, Dallas, TX, United States; ^9^ Department of Urology, Second Faculty of Medicine, Charles University, Prag, Czechia; ^10^ Institute for Urology and Reproductive Health, I.M. Sechenov First Moscow State Medical University, Moscow, Russia; ^11^ Division of Urology, Department of Special Surgery, Jordan University Hospital, The University of Jordan, Amman, Jordan

**Keywords:** prostate cancer, salvage radical prostatectomy, race, cancer specific survival, ethnicity, post-radiotherapy recurrence

## Abstract

**Background:**

To test the effect of race/ethnicity on cancer-specific mortality (CSM) after salvage radical prostatectomy (SRP).

**Material and methods:**

We relied on the Surveillance, Epidemiology and End Results database (SEER, 2004–2016) to identify SRP patients of all race/ethnicity background. Univariate and multivariate Cox regression models addressed CSM according to race/ethnicity.

**Results:**

Of 426 assessable SRP patients, Caucasians accounted for 299 (69.9%) vs. 68 (15.9%) African-Americans vs. 39 (9.1%) Hispanics vs. 20 (4.7%) Asians. At diagnosis, African-Americans (64 years) were younger than Caucasians (66 years), but not younger than Hispanics (66 years) and Asians (67 years). PSA at diagnosis was significantly higher in African-Americans (13.2 ng/ml), Hispanics (13.0 ng/ml), and Asians (12.2 ng/ml) than in Caucasians (7.8 ng/ml, p = 0.01). Moreover, the distribution of African-Americans (10.3%–36.6%) and Hispanics (0%–15.8%) varied according to SEER region. The 10-year CSM was 46.5% in African-Americans vs. 22.4% in Caucasians vs. 15.4% in Hispanics vs. 15.0% in Asians. After multivariate adjustment (for age, clinical T stage, lymph node dissection status), African-American race/ethnicity was an independent predictor of higher CSM (HR: 2.2, p < 0.01), but not Hispanic or Asian race/ethnicity. The independent effect of African-American race/ethnicity did not persist after further adjustment for PSA.

**Conclusion:**

African-Americans treated with SRP are at higher risk of CSM than other racial/ethnic groups and also exhibited the highest baseline PSA. The independent effect of African-American race/ethnicity on higher CSM no longer applies after PSA adjustment since higher PSA represents a distinguishing feature in African-American patients.

## Introduction

Salvage radical prostatectomy is rarely used, even though guidelines recommend it in select patients (1, 2). No historical epidemiological studies addressed salvage radical prostatectomy patients with respect to the importance of race/ethnicity. In consequence, the effect of race/ethnicity is unknown with respect to patient characteristics at diagnosis and its effect on cancer-specific mortality (CSM). It is currently under debate, whether African-American race/ethnicity is associated with adverse characteristics at diagnosis, as well as after treatment, and multiple studies continue to fuel that debate (3–11). However, no such debate exists in the context of salvage radical prostatectomy based on extreme rarity of studies that addressed this topic.

To address this void, we tested the effect of race/ethnicity on patient and tumor characteristics at diagnosis, as well as on CSM. We relied on the Surveillance, Epidemiology and End Results (SEER) database 2004–2016. Race/ethnicity was defined as Caucasians vs. African-Americans vs. Hispanics vs. Asians.

## Material and methods

### Study population

SEER is a database which samples cancer statistics within the United States. The current SEER database includes approximately 35% of the US population and approximates it in demographic composition and cancer incidence. Within the SEER database (2004−2016), we identified patients ≥18 years old with histologically confirmed adenocarcinoma of the prostate, diagnosed at biopsy (International Classification of Disease for Oncology [ICD-O-3] code 8140 site code C61.9) (12). Race/ethnicity was defined as either Caucasian, African American, Hispanic, or Asian. SEER regions were defined as West (Registries Los Angeles, New Mexico, San-Jose-Monterey, Seattle, California, San Francisco-Oakland, Utah, Alaska, Hawaii) vs. Midwest (Registries Detroit and Iowa) vs. North-East (Registries Connecticut and New Jersey) vs. South (Registries Atlanta, Louisiana, Rural Georgia, Greater Georgia, Kentucky). Cases identified only at autopsy or death certificate, unknown histology, or non-primary prostate cancer were excluded. Other racial/ethnic groups (Native American, n = 1) or patients with unknown racial/ethnic status (n = 1) were excluded due to small sample size. Salvage radical prostatectomy was defined as radical prostatectomy after prior radiation therapy, as described before (13). PSA value, age, and stage were defined at initial prostate cancer diagnosis. These selection criteria yielded a cohort of 426 assessable salvage radical prostatectomy patients.

### Statistical analysis

The chi-square tested the statistical significance in proportions’ differences. The t-test and Kruskal–Wallis test examined the statistical significance of means’ and distributions’ differences.

Kaplan–Meier plots and univariate and multivariate Cox regression models after adjustment for age, PSA, clinical T stage, and lymph node dissection status tested the effect of race/ethnicity on salvage radical prostatectomy patients. All tests were two sided with a level of significance set at p < 0.05, and R software environment for statistical computing and graphics (version 3.4.3) was used for all analyses (14).

## Results

### Descriptive characteristics of the study population

**Figure 1:**
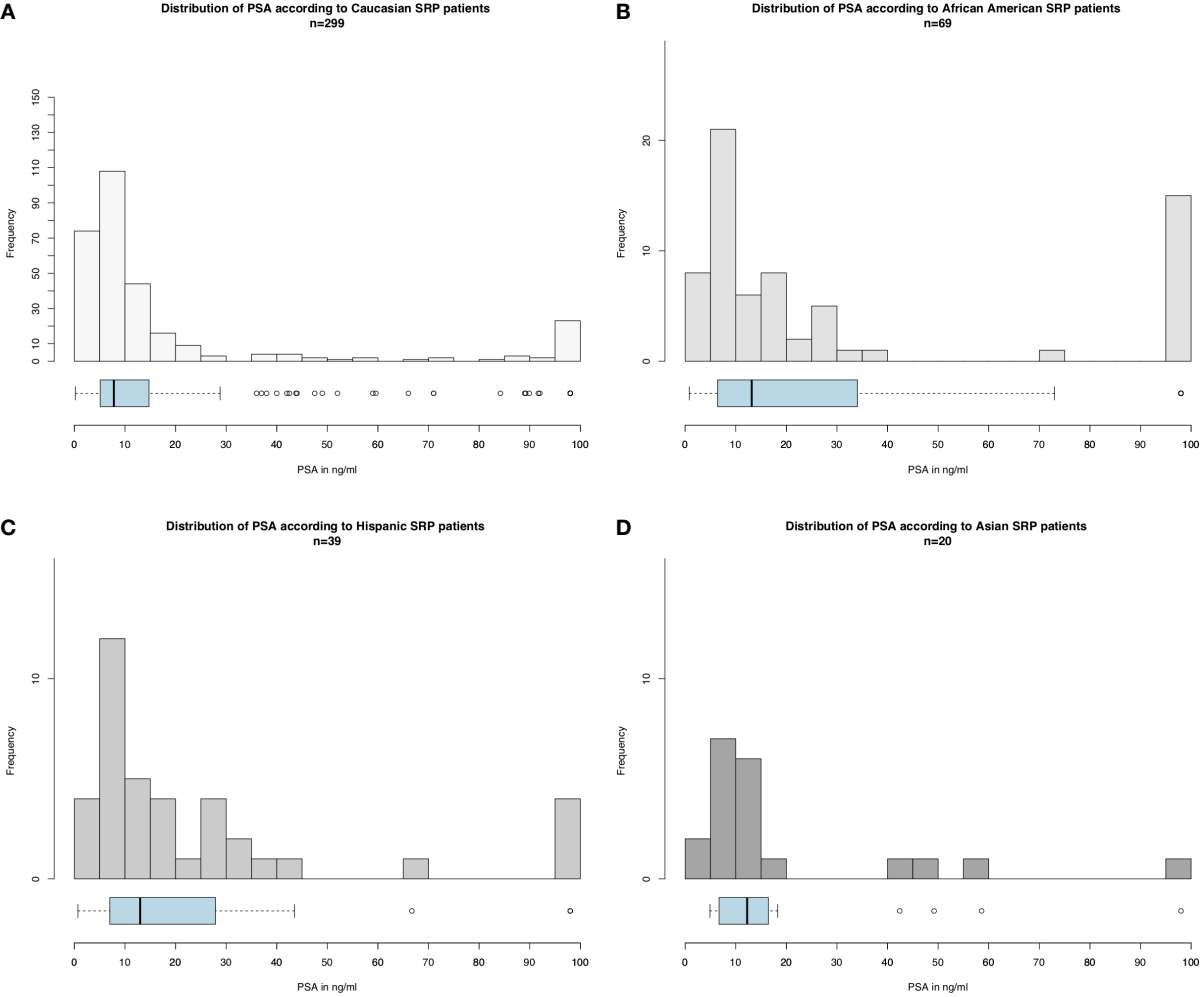
Histograms and boxplot and whisker plots depicting baseline PSA distribution according to race/ethnicity in salvage radical prostatectomy patients for **(A)** Caucasians, **(B)** African-Americans, **(C)** Hispanics, and **(D)** Asians (please be aware of different scales of the Y-axis).

Of 426 salvage radical prostatectomy patients (**Table 1**), Caucasians accounted for 299 (70.2%) vs. 68 (16.0%) African-Americans vs. 39 (9.2%) Hispanics vs. 20 (4.7%) Asians. At diagnosis, African-Americans were younger (64 years [IQR 58–72]) than Caucasians (66 years [IQR 61–74], p = 0.046), but not Hispanics (66 years [IQR 59–74], p = 0.4) or Asians (67 years [IQR 65–71], p = 0.6). PSA at diagnosis (**Figure 1**) was significantly higher in African-Americans (13.2 ng/ml [IQR 6.6–32.8]), Hispanics (13.0 ng/ml [IQR 7.0–27.9]), and Asians (12.2 ng/ml [IQR 6.8–15.5]), than in Caucasians (7.8 ng/ml [IQR 5.1–14.8], p < 0.01). No clinically meaningful or statistically significant race/ethnic differences were recorded in the clinical T stage at diagnosis, biopsy Gleason score, Gleason score at salvage radical prostatectomy, pathological T stage, as well as rate of lymph node dissection.

**Table 1:** Descriptive characteristics of 426 salvage radical prostatectomy patients, stratified according to race/ethnicity, namely, Caucasians, African Americans, Hispanics, and Asians, diagnosed within the Surveillance, Epidemiology, and End Results database from 2004 to 2016.

Variable		Overall n = 426	Caucasiann = 299 (70.2%)	African-Americann = 68 (16.0%)	Hispanic n=39 (9.2%)	Asian n=20 (4.7%)	p value
**Age at diagnosis (year)**	Median (IQR)	66 (61-73)	66 (61-74)	64 (58-72)	66 (59-74)	67 (65-71)	0.1
**Follow up (months)**	Median (IQR)	75 (31-115)	76 (32-116)	71 (24-106)	53 (33-113)	97 (40-123)	0.7
**PSA (ng/ml)**	Median (IQR)	8.8 (5.4-18.5)	7.8 (5.1-14.8)	13.2 (6.6-32.8)	13.0 (7.0-27.9)	12.2 (6.8-15.5)	0.01
**Gleason score in biopsy**	≤6	45 (10.6)	35 (11.7)	6 (8.8)	3 (7.7)	1 (5.0)	0.9
	7	57 (13.4)	40 (13.4)	9 (13.2)	6 (15.4)	2 (10.0)	
	8-10	61 (14.3)	45 (15.1)	9 (13.2)	3 (7.7)	4 (20.0)	
	Unknown	263 (61.7)	179 (59.9)	44 (64.7)	27 (69.2)	13 (65.0)	
**Gleason score in RP**	≤6	14 (3.3)	12 (4)	1 (1.5)	1 (2.6)	0 (0)	0.1
	7	18 (4.2)	13 (4.3)	0 (0)	3 (7.7)	2 (10.0)	
	8-10	17 (4.0)	13 (4.3)	1 (1.5)	1 (2.6)	2 (10.0)	
	Unknown	377 (88.5)	261 (87.3)	66 (97.1)	34 (87.2)	16 (80.0)	
**cT stage**	T1	205 (48.1)	143 (47.8)	36 (52.9)	15 (38.5)	11 (55.0)	0.5
	T2	149 (35)	108 (36.1)	20 (29.4)	13 (33.3)	8 (40.0)	
	T3	25 (5.9)	19 (6.4)	3 (4.4)	3 (7.7)	0 (0)	
	T4	18 (4.2)	13 (4.3)	3 (4.4)	2 (5.1)	0 (0)	
	Tx	29 (6.8)	16 (5.4)	6 (8.8)	6 (15.4)	1 (5.0)	
**pT stage**	T2	101 (23.7)	73 (24.4)	12 (17.6)	7 (17.9)	9 (45.0)	0.048
	T3	43 (10.1)	32 (10.7)	6 (8.8)	3 (7.7)	2 (10.0)	
	T4	4 (0.9)	2 (0.7)	0 (0)	2 (5.1)	0 (0)	
	Tx	278 (65.3)	192 (64.2)	50 (73.5)	27 (69.2)	9 (45.0)	
**LND**	Not performed	305 (71.6)	219 (73.2)	50 (73.5)	25 (64.1)	11 (55.0)	0.3
	performed	120 (28.2)	80 (26.8)	17 (25)	14 (35.9)	9 (45.0)	
	Unknown	1 (0.3)	0 (0)	1 (1.5)	0 (0)	0 (0)	
**Number of removed lymph nodes**	Median (IQR)	7 (3-11)	7 (4-13)	4 (3-8)	8 (4-11)	8 (3-11)	0.3
**pN stage**	pN0	97 (22.8)	65 (21.7)	14 (20.6)	11 (28.2)	7 (35.0)	0.6
	pN1	27 (6.3)	18 (6)	3 (4.4)	4 (10.3)	2 (10.0)	
	pNx	302 (70.9)	216 (72.2)	51 (75.0)	24 (61.5)	11 (55.0)	
**Marital status**	Married	282 (66.2)	214 (71.6)	34 (50.0)	23 (59.0)	11 (55.0)	<0.01
	Unmarried	111 (26.1)	64 (21.4)	28 (41.2)	12 (30.8)	7 (35.0)	
	Unknown	33 (7.7)	21 (7.0)	6 (8.8)	4 (10.2)	2 (10.0)	
**Region**	West	180 (42.3)	122 (40.8)	17 (25.0)	26 (66.7)	15 (75.0)	<0.001
	Midwest	41 (9.6)	26 (8.7)	15 (22.1)	0 (0)	0 (0)	
	North-East	93 (21.8)	69 (23.1)	11 (16.2)	10 (25.6)	3 (15.0)	
	South	112 (26.3)	82 (27.4)	25 (36.8)	3 (7.7)	2 (10.0)	
**Rural/urban**	Rural	43 (10.1)	36 (12)	4 (5.9)	3 (7.7)	0 (0)	0.2
	Urban	383 (89.9)	263 (88)	64 (94.1)	36 (92.3)	20 (100)	
**Socioeconomic status**	1st quartile	115 (27.0)	98 (32.8)	14 (20.6)	2 (5.1)	1 (5.0)	<0.001
	2nd–4th quartile	311 (73.0)	201 (67.2)	54 (79.4)	37 (94.9)	19 (95.0)	
**Type of radiotherapy**	EBRT	314 (73.7)	211 (70.6)	51 (75.0)	38 (97.4)	14 (70.0)	0.01
	BT	67 (15.7)	53 (17.7)	8 (11.8)	1 (2.6)	5 (25.0)	
	BT+EBRT	45 (10.6)	35 (11.7)	9 (13.2)	0 (0)	1 (5.0)	

### Regional and patient characteristic differences according to race/ethnicity in salvage radical prostatectomy

**Figure 2:**
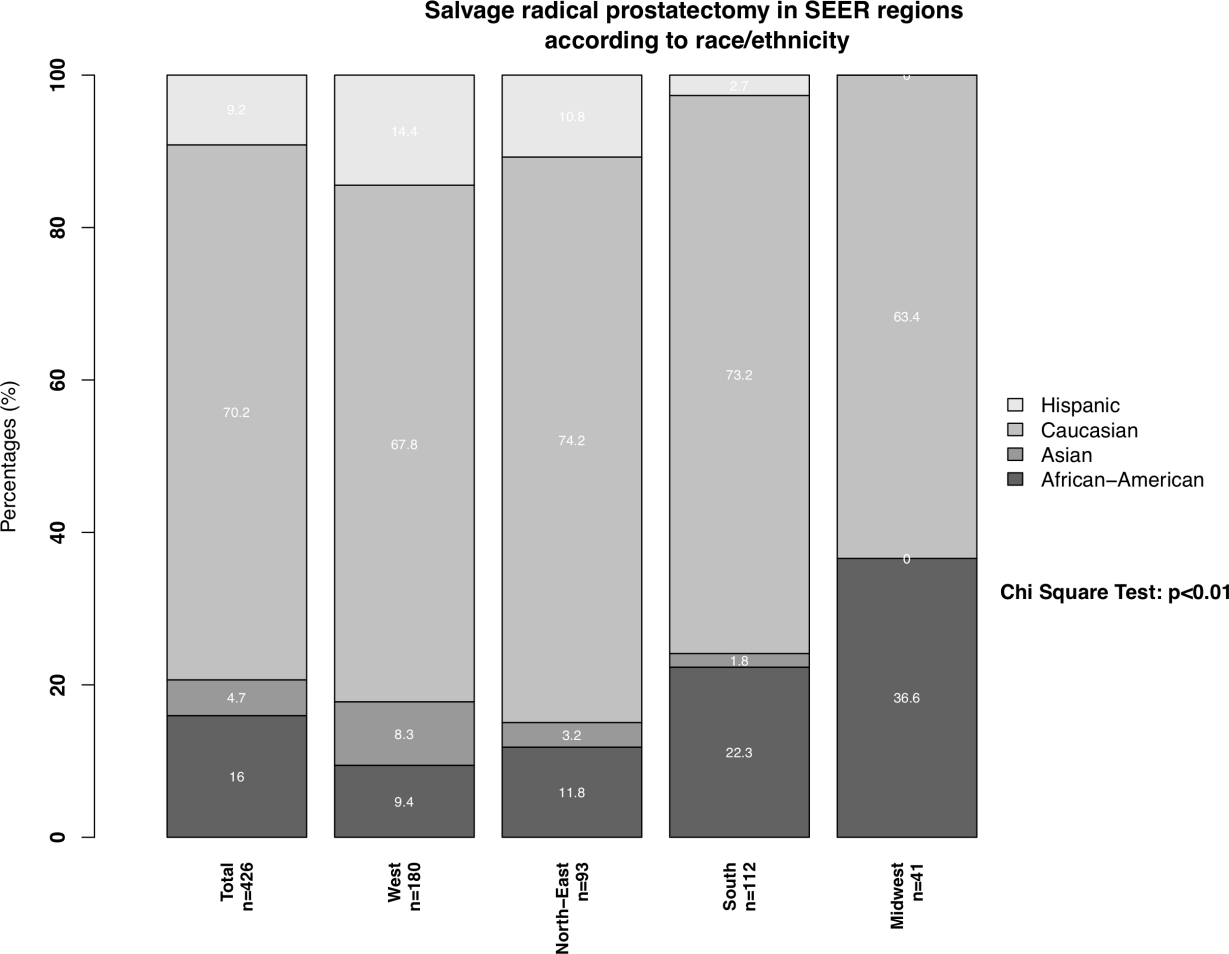
Stacked barplots depicting SEER region distribution according to African-Americans, Caucasians, Hispanics, and Asians, who underwent salvage radical prostatectomy. PCa: prostate cancer.

Important regional differences were observed in the distribution of salvage radical prostatectomy patients according to race/ethnicity (**Figure 2**). First, the proportions of African-Americans, Hispanics, and Asians who underwent salvage radical prostatectomy significantly differed across SEER regions (all p < 0.02). For example, in the West, the proportions of African-American, Hispanic, and Asian men who underwent salvage radical prostatectomy were respectively 9.4%, 14.4%, and 8.3%. Conversely, in the Midwest, African-Americans, Hispanics, and Asians accounted for 36.6%, 0%, and 0% of all salvage radical prostatectomies.

### CSM and OCM in salvage radical prostatectomy according to race/ethnicity

**Figure 3:**
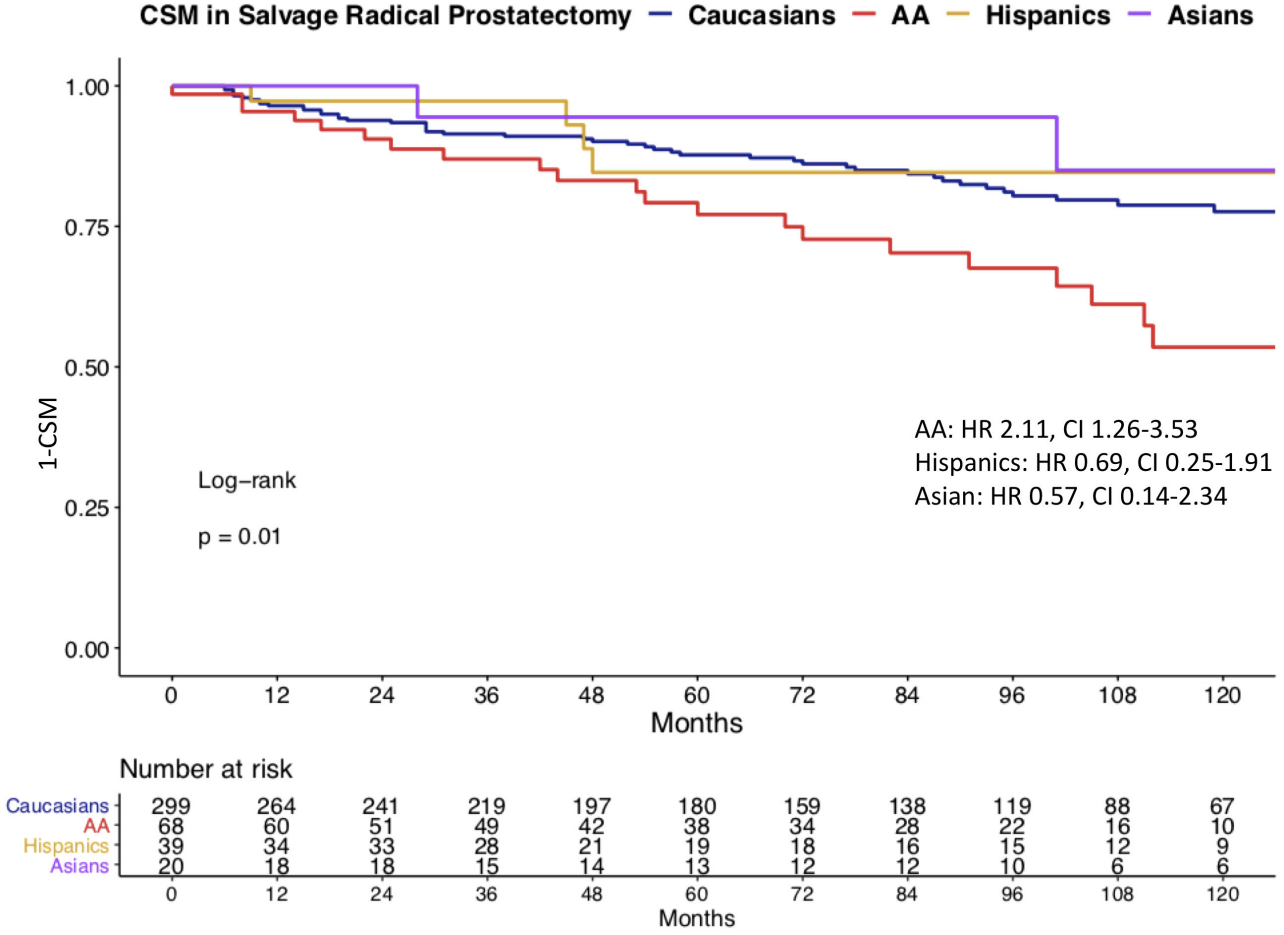
Kaplan–Meier plot illustrating unadjusted cancer-specific mortality (CSM) in salvage radical prostatectomy patients, according to racial/ethnic groups. CSM: cancer-specific mortality, AA: African-American, HR: hazard ratio, CI: confidence interval.

We observed important CSM and other-cause mortality (OCM) differences in salvage radical prostatectomy patients according to race/ethnicity (**Figure 3**). Specifically, the 10-year CSM was 46.5% in African-Americans vs. 22.4% in Caucasians vs. 15.4% in Hispanics vs. 15.0% in Asians. After multivariate adjustment (**Table 2**) for tumor and patient characteristics (age, clinical T stage, and lymph node dissection status), African-American race/ethnicity was an independent predictor of higher CSM (hazard ratio [HR] 2.15, confidence interval [CI] 1.26–3.66, p < 0.01), but not Hispanic (HR 0.46, CI 0.16–1.30, p = 0.1) or Asian (HR 0.83, CI 0.20–3.44, p = 0.8) race/ethnicity. However, the CSM disadvantage in African-Americans disappeared after further multivariate adjustment for PSA (**Table 3**). Finally, we repeated our analyses in matched competing risk regression models and these results virtually perfectly replicated the results based on Cox regression models.

**Table 2:** Univariable and multivariable (after adjustment for age, lymph node dissection status, clinical T stage).

	Univariable analysis			Multivariable analysis		
	HR	95% CI	P-value	HR	95% CI	P-value
**Race**						
**Caucasian**	1 (Ref)	–	–	1 (Ref)	–	–
**African-American**	2.11	(1.26-3.53)	<0.01	2.15	(1.26-3.66)	<0.01
**Hispanic**	0.69	(0.25-1.91)	0.5	0.46	(0.16-1.30)	0.1
**Asian**	0.57	(0.14-2.36)	0.4	0.83	(0.20-3.44)	0.8
**Age**	1.01	(0.99-1.04)	0.3	1.01	(0.98-1.03)	0.6
**Lymph node dissection**						
**Not performed**	1 (Ref)	–	–	1 (Ref)	–	–
**Performed**	0.35	(0.18-0.67)	<0.001	0.37	(0.19-0.74)	<0.01
**cT1-2**	1 (Ref)	–	–	1 (Ref)	–	–
**cT3-4**	5.75	(3.38-9.85)	<0.001	6.37	(3.65-11.10)	<0.001
**cTx**	7.70	(3.38-17.52)	<0.001	5.88	(2.52-13.69)	<0.001

Cox regression models in salvage radical prostatectomy patients predicting cancer-specific mortality according to race/ethnicity.

**Table 3:** Univariable and multivariable (after adjustment for age, PSA, lymph node dissection status, clinical T stage).

	Univariable analysis			Multivariable analysis		
	HR	95% CI	P-value	HR	95% CI	P-value
**Race**						
**Caucasian**	1 (Ref)	–	–	1 (Ref)	–	–
**African-American**	2.11	(1.26-3.53)	<0.001	1.36	(0.78-2.36)	0.3
**Hispanic**	0.69	(0.25-1.91)	0.5	0.47	(0.17-1.33)	0.2
**Asian**	0.57	(0.14-2.36)	0.4	0.95	(0.23-3.96)	0.9
**Age**	1.01	(0.99-1.04)	0.3	1.01	(0.98-1.04)	0.6
**PSA**	1.03	(1.02-1.04)	<0.001	1.03	(1.02-1.03)	<0.001
**Lymph node dissection**						
**Not performed**	1 (Ref)	–	–	1 (Ref)	–	–
**Performed**	0.35	(0.18-0.67)	<0.001	0.48	(0.24-0.97)	0.04
**cT1-2**	1 (Ref)	–	–	1 (Ref)	–	–
**cT3-4**	5.75	(3.38-9.85)	<0.001	3.44	(1.90-6.24)	<0.001
**cTx**	7.70	(3.38-17.52)	<0.001	1.56	(0.63-3.87)	0.4

Cox regression models in salvage radical prostatectomy patients predicting cancer-specific mortality according to race/ethnicity.

It is of note that OCM demonstrated important variability according to race/ethnicity. Specifically, the 10-year OCM was 24.7% in African-Americans vs. 24.3% in Hispanics vs. 24.0% in Caucasians vs. 0% in Asians.

## Discussion

We hypothesized that differences may exist between racial/ethnic groups according to patient and tumor characteristics, as well as CSM after salvage radical prostatectomy. We tested this hypothesis within the SEER database 2004–2016 and arrived at several noteworthy observations.

First, we identified important differences in patient characteristics in salvage radical prostatectomy patients according to racial/ethnic groups. For example, African-Americans were younger at prostate cancer diagnosis (64 vs. 66 years), relative to Caucasians. Conversely, no age differences were recorded between Caucasians vs. Hispanics and vs. Asians at prostate cancer diagnosis. The age difference was in agreement with previously reported age differences between African-Americans and Caucasians, in the context of primary radical prostatectomy (15–17).

Second, the geographic distribution of salvage radical prostatectomy rates demonstrated important differences, across all race/ethnic groups. Specifically, in African Americans, the rate of salvage radical prostatectomies was lowest in the West (9%) and highest in the Midwest (37%). Conversely, in Caucasians the rate of salvage radical prostatectomies was highest in the North-East and lowest in the Midwest. These observations are in agreement with regional differences in the proportions of African-American patients treated for primary prostate cancer (18, 19). Moreover, these observations may imply that African-Americans may be given higher priority for salvage radical prostatectomy in the Midwest than in the West. However, this interpretation is subject to bias due to small patient number. Moreover, our findings cannot be compared to other studies since no previous population-based studies formally addressed the geographic distribution of salvage radical prostatectomy patients. However, previous studies addressing differences in treatment of intermediate-risk prostate cancer according to racial/ethnic differences of all SEER regions indicated that these differences disappear after adjustment for baseline prostate cancer characteristics. Specifically, the authors therefore hypothesized that differences cannot be exclusively explained by differences in access to health care system of specific racial/ethnic groups or rural geographical areas (20, 21). However, these analyses have never been conducted for SRP patients and should be subject of further research.

Third, we examined baseline prostate cancer characteristics according to racial/ethnic groups. Median PSA as well as the entire distribution of the PSA values was higher in African-Americans than in all three other race/ethnic groups. The baseline PSA disadvantage observed in salvage radical prostatectomy African-American patients relative to Caucasians has previously been reported in the context of primary radical prostatectomy (22–24). Despite having higher PSA baseline values, African-Americans exhibited marginally lower rates of pathologically non-organ confined stage than Caucasians. However, this observation needs to be interpreted in the light of a very elevated rate of missing stage information in all race/ethnic groups. The rate of missing data was highest in pathological Gleason score, pathological T stage, Gleason score at biopsy, and clinical T stage, in that order. Conversely, baseline PSA values were available for all assessable salvage radical prostatectomy patients. In consequence, baseline PSA value disadvantage observed in African-Americans is more reliable and robust than the information derived from stage and grade at biopsy (missing information 5.05%–15.4% and 59.9%–69.2%) or pathologic stage and grade at salvage radical prostatectomy (missing information 45.0%–73.5% and 80.0%–97.1%). The observed rates of missing values in the current study exceed the rates of missing values in institutional salvage radical prostatectomy series. Nonetheless, institutional salvage radical prostatectomy series were affected by missing value rates that significantly exceeded missing value rates applicable to primary radical prostatectomy (25, 26). In consequence, biases related to missing information are universally applicable to all salvage radical prostatectomy series. Nonetheless, it should be emphasized that population-derived data, such as the current SEER database, are more heavily affected by missing data than institutional series.

Finally, we investigated CSM rates according to race/ethnicity. To allow comparability with previous studies, we relied on Cox regression models (27–29). In univariate Cox regression models, African-Americans exhibited a 2.1-fold higher CSM. It is of note that OCM was comparable between Caucasians and African-American salvage radical prostatectomy patients. This observation is very different from OCM rates in African-Americans reported after primary radical prostatectomy patients (30). Specifically, these rates were significantly higher in African-Americans than in Caucasians. Taken together, these observations imply that the selection criteria based on comorbidities may predispose to higher OCM in Caucasian, African American, and Hispanic salvage radical prostatectomy patients. After multivariate adjustment for patient age, clinical T stage, and lymph node dissection status, African American race/ethnicity achieved independent predictor status for higher CSM. Specifically, African-Americans exhibited a 2.2-fold higher CSM rate than Caucasians. However, after further adjustment for PSA at diagnosis, this CSM difference disappeared. This observation implies that the PSA disadvantage at baseline is inherent to African-American patients. Indeed, we illustrated very important and statistically significant differences in PSA distribution in African-American and other racial/ethnic groups, predominantly Caucasians (**Figure 1**). In consequence, adjustment for PSA values, the main distinguishing feature of African-American salvage radical prostatectomy patients, should be interpreted as overfitting. Under this premise, multivariable findings without PSA adjustment represent a more objective assessment of the effect of race/ethnicity on CSM, since cT stage and performance of lymph node dissection also have an even higher positive/negative effect on CSM than the PSA. Moreover, as stated in the EAU guidelines, predominantly PSA at prostate cancer recurrence prior to a possible performance of SRP should be used for classification (31–33). Unfortunately, these data are not available in the SEER database. Nonetheless, to the best of our knowledge, no previous study examined baseline PSA or subsequent PSA profiles of salvage radical prostatectomy patients, relative to Caucasians or other racial/ethnic groups. In consequence, our observations cannot be directly compared to the findings of others. It is also of interest that the PSA profiles of Hispanics and Asians were moderately higher than that of Caucasians. However, the importance of these observations is not comparable to that of African-Americans, since CSM reported in Hispanics and Asians does not differ from that of Caucasians.

Taken together, our observations indicate that salvage radical prostatectomy proportions significantly differ between SEER regions according to race/ethnicity. Moreover, baseline patient age and PSA baseline characteristics also differ according to race/ethnicity. Specifically, significantly higher PSA values are associated with African-American race/ethnicity. Moreover, African-American race/ethnicity is also associated with higher CSM. This association is based on the unfavorable PSA profile of African-American patients that this is inherent to this racial/ethnic group. In consequence, the PSA profile should not be dissociated from race/ethnicity.

Our work has limitations and should be interpreted in the context of its retrospective and population-based design with its associated limitations (34). Moreover, the SEER database provides no information on age, longitudinal PSA values, repeat biopsy findings, or time interval between radiotherapy and salvage radical prostatectomy in patients with recurrent prostate cancer, as well as on metastatic progression. Similarly, additional treatment information is limited, and especially androgen deprivation therapy status is unknown. Finally, despite the very large prostate cancer patient population of the SEER database, the sample of salvage radical prostatectomy patients is relatively small. The sample size limitation undermines the statistical significance of some comparisons. However, our cohort is the largest ever reported salvage radical prostatectomy cohort relative to other studies that addressed oncological outcomes after salvage radical prostatectomy and consisted of up to 404 patients (22). Interestingly, this multi-institutional cohort as well as the majority of other institution data focused on biochemical recurrence rates and addressed cohorts that ranged from 32 to 55 patients (23–25, 35, 36). Our cohort relies on a small sample that resulted in lack of significant differences in some subgroup comparisons. However, it should be emphasized that the SEER database is designed with the intent of providing proportional representation of the US population. In consequence, few if any other databases will provide a larger sample of those salvage radical prostatectomy patients according to racial/ethnic groups.

## Data availability statement

The raw data supporting the conclusions of this article will be made available by the authors, without undue reservation.

## Ethics statement

Ethical review and approval was not required for the study on human participants in accordance with the local legislation and institutional requirements. Written informed consent for participation was not required for this study in accordance with the national legislation and the institutional requirements.

## Author contributions

Conceptualization: MW, CW, FC, and PK; Data curation: MW, CC, and ZT; Formal analysis: MW, CW, and ZT; Investigation: MW, CW, CC, and LN; Methodology: MW, ZT, FP, and BH; Project administration resources. SEER database software R system; Supervision: PK, FS, and FP; Validation: ZT, PK, SS, FS, ABr, MG, ABe, PM, and FC. All authors contributed to the article and approved the submitted version.

## Conflict of interest

The authors declare that the research was conducted in the absence of any commercial or financial relationships that could be construed as a potential conflict of interest.

## Publisher’s note

All claims expressed in this article are solely those of the authors and do not necessarily represent those of their affiliated organizations, or those of the publisher, the editors and the reviewers. Any product that may be evaluated in this article, or claim that may be made by its manufacturer, is not guaranteed or endorsed by the publisher.

## References

[1] Mottet N Cornford P Van den Bergh RCN Briers E De Santis M Fanti S. EAU - EANM - ESTRO - ESUR - SIOG. guidelines on prostate cancer. Eur Urol (2020). 10.1016/j.eururo.2020.09.046 33039206

[2] Mohler JL Srinvicas S Antonarakis ES Armstrong AJ Bekelman JE Cheng H. Prostate cancer, version 1.2020, NCCN clinical practice guidelines in oncology. J Natl Compr Canc Netw (2020).

[3] Schmid M Meyer CP Reznor G Choueiri TK Hanske J Sammon JD. Racial differences in the surgical care of Medicare beneficiaries with localized prostate cancer. JAMA Oncol (2016) 2(1):85–93. doi: 10.1001/jamaoncol.2015.3384 26502115 PMC5018381

[4] Mahal BA Aizer AA Ziehr DR Hyatt AS Sammon JD Schmid M. Trends in disparate treatment of African American men with localized prostate cancer across national comprehensive cancer network risk groups. Urology (2014) 84(2):386–92. doi: 10.1016/j.urology.2014.05.009 24975710

[5] Würnschimmel C Nocera L Wenzel M Ruvolo CC Tian Z Saad F. Race/Ethnicity may be an important predictor of life expectancy in localized prostate cancer patients: Novel analyses using social security administration life tables. J Racial Ethn Health Disparities (2022). doi: 10.1007/s40615-022-01257-y PMC9988799 35182370

[6] Würnschimmel C Wenzel M Chierigo F Flammia RS Tian Z Saad F. External beam radiotherapy and radical prostatectomy are associated with better survival in Asian prostate cancer patients. Int J Urol (2022) 29(1):17–24. doi: 10.1111/iju.14701 34553428

[7] Krimphove MJ Cole AP Fletcher SA Harmouch SS Berg S Lipsitz SR. Evaluation of the contribution of demographics, access to health care, treatment, and tumor characteristics to racial differences in survival of advanced prostate cancer. Prostate Cancer Prostatic Dis (2019) 22(1):125–36. doi: 10.1038/s41391-018-0083-4 30171227

[8] Würnschimmel C Wenzel M Collà Ruvolo C Nocera L Tian Z Saad F. Life expectancy in metastatic prostate cancer patients according to racial/ethnic groups. Int J Urol (2021) 28(8):862–9. doi: 10.1111/iju.14595 33993551

[9] Nocera L Wenzel M Collà Ruvolo C Würnschimmel C Tian Z Gandaglia G. The effect of race/ethnicity on active treatment rates among septuagenarian or older low risk prostate cancer patients. Urol Oncol (2021) 39(11):785.e11–785.e17. doi: 10.1016/j.urolonc.2021.04.004 33992522

[10] Dess RT Hartman HE Mahal BA Soni PD Jackson WC Cooperberg MR. Association of black race with prostate cancer-specific and other-cause mortality. JAMA Oncol (2019) 5(7):975–83. doi: 10.1001/jamaoncol.2019.0826 PMC6547116 31120534

[11] Würnschimmel C Wenzel M Collà Ruvolo C Nocera L Tian Z Saad F. Survival advantage of Asian metastatic prostate cancer patients treated with external beam radiotherapy over other races/ethnicities. World J Urol (2021) 39(10):3781–7. doi: 10.1007/s00345-021-03720-7 PMC8519889 33978812

[12] About the SEER program. SEER. Available at: https://seer.cancer.gov/about/overview.html.

[13] Pokala N Huynh DL Henderson AA Johans C. Survival outcomes in men undergoing radical prostatectomy after primary radiation treatment for adenocarcinoma of the prostate. Clin Genitourin Cancer (2016) 14(3):218–25. doi: 10.1016/j.clgc.2015.12.010 26774347

[14] RCT. R: A language and environment for statistical computing (2017). Available at: https://wwwr-projectorg2017.

[15] Preisser F Nazzani S Bandini M Marchioni M Tian Z Saad F. Racial disparities in lymph node dissection at radical prostatectomy: A surveillance, epidemiology and end results database analysis. Int J Urol (2018) 25(11):929–36. doi: 10.1111/iju.13780 30146729

[16] Jeong IG Dajani D Verghese M Hwang J Cho YM Hong JH. Differences in the aggressiveness of prostate cancer among Korean, Caucasian, and African American men: A retrospective cohort study of radical prostatectomy. Urol Oncol (2016) 34(1):3.e9–14. doi: 10.1016/j.urolonc.2015.08.004 26345648

[17] Amini E Palmer TC Cai J Lieskovsky G Daneshmand S Djaladat H. Association between race and oncologic outcome following radical prostatectomy for clinically organ-confined prostate cancer: a long-term follow-up study. World J Urol (2018) 36(8):1233–9. doi: 10.1007/s00345-018-2266-y 29536157

[18] Harlan L Brawley O Pommerenke F Wali P Kramer B. Geographic, age, and racial variation in the treatment of local/regional carcinoma of the prostate. J Clin Oncol (1995) 13(1):93–100. doi: 10.1200/JCO.1995.13.1.93 7799048

[19] Kamel MH Bimali M Khalil MI Eltahawy E Su LJ Bissada NK. Regional trends in average years of potential life lost (AYPLL) secondary to prostate cancer deaths among caucasians and African americans treated by surgery or radiation. Int Urol Nephrol (2019) 51(4):561–9. doi: 10.1007/s11255-019-02116-2 30840195

[20] Wenzel M Nocera L Ruvolo CC Würnschimmel C Tian Z Shariat SF. Racial/Ethnic disparities in tumor characteristics and treatments in favorable and unfavorable intermediate risk prostate cancer. J Urol (2021) 206(1):69–79. doi: 10.1097/JU.0000000000001695 33683934

[21] Wenzel M Collà Ruvolo C Nocera L Würnschimmel C Tian Z Shariat SF. Regional differences in patient age and prostate cancer characteristics and rates of treatment modalities in favorable and unfavorable intermediate risk prostate cancer across united states SEER registries. Cancer Epidemiol (2021) 74:101994. doi: 10.1016/j.canep.2021.101994 34364187

[22] Nocera L Wenzel M Collà Ruvolo C Würnschimmel C Tian Z Gandaglia G. The impact of race/ethnicity on upstaging and/or upgrading rates among intermediate risk prostate cancer patients treated with radical prostatectomy. World J Urol (2022) 40(1):103–10. doi: 10.1007/s00345-021-03816-0 34436637

[23] Velasquez MC Chinea FM Kwon D Prakash NS Barboza MP Gonzalgo ML. The influence of ethnic heterogeneity on prostate cancer mortality after radical prostatectomy in Hispanic or Latino men: A population-based analysis. Urology (2018) 117:108–14. doi: 10.1016/j.urology.2018.03.036 PMC6062850 29630954

[24] Powell IJ Dyson G Land S Ruterbusch J Bock CH Lenk S. Genes associated with prostate cancer are differentially expressed in African American and European American men. Cancer Epidemiol Biomarkers Prev (2013) 22(5):891–7. doi: 10.1158/1055-9965.EPI-12-1238 PMC4097306 23515145

[25] Sundi D Ross AE Humphreys EB Han M Partin AW Carter HB. African American Men with very low-risk prostate cancer exhibit adverse oncologic outcomes after radical prostatectomy: should active surveillance still be an option for them? J Clin Oncol (2013) 31(24):2991–7. doi: 10.1200/JCO.2012.47.0302 PMC3739860 23775960

[26] Heidenreich A Ohlmann C Ozgür E Engelmann U. [Functional and oncological outcome of salvage prostatectomy of locally recurrent prostate cancer following radiation therapy]. Urologe A (2006) 45(4):474–81. doi: 10.1007/s00120-006-0995-9 16465521

[27] Chade DC Shariat SF Cronin AM Savage CJ Karnes RJ Blute ML. Salvage radical prostatectomy for radiation-recurrent prostate cancer: a multi-institutional collaboration. Eur Urol (2011) 60(2):205–10. doi: 10.1016/j.eururo.2011.03.011 PMC3124574 21420229

[28] Sanderson KM Penson DF Cai J Groshen S Stein JP Lieskovsky G. Salvage radical prostatectomy: quality of life outcomes and long-term oncological control of radiorecurrent prostate cancer. J Urol (2006) 176(5):2025–31. doi: 10.1016/j.juro.2006.07.075 17070244

[29] Mandel P Steuber T Ahyai S Kriegmair M Schiffmann J Boehm K. Salvage radical prostatectomy for recurrent prostate cancer: verification of European association of urology guideline criteria. BJU Int (2016) 117(1):55–61. doi: 10.1111/bju.13103 25711672

[30] Bandini M Preisser F Nazzani S Marchioni M Tian Z Mazzone E. The effect of other-cause mortality adjustment on access to alternative treatment modalities for localized prostate cancer among African American patients. Eur Urol Oncol (2018) 1(3):215–22. doi: 10.1016/j.euo.2018.03.007 31102624

[31] Preisser F Würnschimmel C Pose RM Heinze A Steuber T Michl U. Concordance of biopsy and pathologic ISUP grading in salvage radical prostatectomy patients for recurrent prostate cancer. Prostate (2022) 82(2):254–9. doi: 10.1002/pros.24268 34807461

[32] Wenzel M Würnschimmel C Nocera L Collà Ruvolo C Tian Z Shariat SF. Salvage radical prostatectomy: Baseline prostate cancer characteristics and survival across SEER registries. Clin Genitourin Cancer (2021) 19(4):e255–63. doi: 10.1016/j.clgc.2021.03.015 33849813

[33] Wenzel M Würnschimmel C Nocera L Collà Ruvolo C Tian Z Shariat SF. The effect of lymph node dissection on cancer-specific survival in salvage radical prostatectomy patients. Prostate (2021) 81(6):339–46. doi: 10.1002/pros.24112 33666271

[34] Noone A-M Lund JL Mariotto A Cronin K McNeel T Deapen D. Comparison of SEER treatment data with Medicare claims. Med Care (2016) 54(9):e55–64. doi: 10.1097/MLR.0000000000000073 PMC4981219 24638121

[35] Leonardo C Simone G Papalia R Franco G Guaglianone S Gallucci M. Salvage radical prostatectomy for recurrent prostate cancer after radiation therapy. Int J Urol (2009) 16(6):584–6. doi: 10.1111/j.1442-2042.2008.02209.x 19453762

[36] Heidenreich A Richter S Thüer D Pfister D. Prognostic parameters, complications, and oncologic and functional outcome of salvage radical prostatectomy for locally recurrent prostate cancer after 21st-century radiotherapy. Eur Urol (2010) 57(3):437–43. doi: 10.1016/j.eururo.2009.02.041 19303197

